# Dissecting the membrane-microtubule sensor in grapevine defence

**DOI:** 10.1038/s41438-021-00703-y

**Published:** 2021-12-01

**Authors:** Pingyin Guan, Wenjing Shi, Michael Riemann, Peter Nick

**Affiliations:** 1 College of Horticulture, China Agricultural University, Beijing, 100193 China; 2 Molecular Cell Biology, Botanical Institute, Karlsruhe Institute of Technology, Fritz-Haber-Weg 4, 76131 Karlsruhe, Germany

**Keywords:** Plant cytoskeleton, Biotic

## Abstract

Specific populations of plant microtubules cooperate with the plasma membrane to sense and process abiotic stress signals, such as cold stress. The current study derived from the question, to what extent this perception system is active in biotic stress signalling. The experimental system consisted of grapevine cell lines, where microtubules or actin filaments are visualised by GFP, such that their response became visible in vivo. We used the bacterial elicitors harpin (inducing cell-death related defence), or flg22 (inducing basal immunity) in combination with modulators of membrane fluidity, or microtubules. We show that DMSO, a membrane rigidifier, can cause microtubule bundling and trigger defence responses, including activation of phytoalexin transcripts. However, DMSO inhibited the gene expression in response to harpin, while promoting the gene expression in response to flg22. Treatment with DMSO also rendered microtubules more persistent to harpin. Paradoxically, Benzylalcohol (BA), a membrane fluidiser, acted in the same way as DMSO. Neither GdCl_3_, nor diphenylene iodonium were able to block the inhibitory effect of membrane rigidification on harpin-induced gene expression. Treatment with taxol stabilised microtubule against harpin but amplified the response of PAL transcripts. Therefore, the data support implications of a model that deploys specific responses to pathogen-derived signals.

## Introduction

Plant basal immunity is generally activated through the perception of pathogen-associated molecular patterns (PAMPs) by plasma-membrane localised pattern-recognition receptors (PRRs), and therefore designated as PAMP-triggered immunity (PTI)^1–3^. During a second round of evolutionary warfare, several pathogens have evolved the ability to breach the immunity of their host by secreting specific molecules, termed effectors, either to evade detection, or to suppress PTI, leading to effector-triggered susceptibility (ETS) of the host^4^. Subsequently, in consequence of prolonged co-evolution with the pathogen, some host species have acquired means to detect these effectors and re-install a second level of defence, effector-triggered immunity (ETI). In this context, intracellular nucleotide-binding site-leucine-rich repeat (LRR) receptors (NLRs), which genetically become manifest as *Resistance (R)* loci play an important role^5^. Although PTI and ETI represent different layers of defence, transcriptomic analyses revealed that PAMP-responsive transcripts overlap with ETI-related transcripts to a large extent^6^, indicating that PTI and ETI share a part of the signalling pathway. Likewise, many of the cellular events upstream of gene expressions, such as ion fluxes across the plasma membrane, apoplastic respiratory burst, cytoskeletal remodelling, or activation of mitogen-activated protein kinase (MAPK) cascades, seem to be shared. This was concluded from a comparative study in grapevine cells, where responses to the bacterial elicitors flg22 (triggering PTI) and harpin (triggering a cell-death related defence response) were compared along with the induction of different defence genes^7^. The defence has to initiate at the site, where the pathogen tries to invade, at the periphery of the cell (i.e. in the cell wall or at the plasma membrane). The signalling input is a mixture of chemical (such as microbial molecules that bind as ligands to receptors) and physical clues (such as perturbations of wall or membrane integrity), which assigns the plasma membrane itself an important role in signalling the plasma membrane itself. However, this role has not attracted the same attention as ligand–receptor interactions and, therefore, has remained somewhat elusive.

The plasma membrane combines long-term stability and short-term dynamics. On the molecular level, three functional components can be discerned^8,9^: the interactions of the lipid bilayer with the subtending cortical cytoskeleton and the cell wall, the interactions between proteins and lipids within the plasma membrane, and the mutual interactions among membrane proteins^8^. All three processes seem to be relevant for defence signalling: receptor proteins located in the plasma membrane are often forming homo- and hetero-oligomers, and the composition of the complex will lead to a different signalling readout. A classic example is the complex between the immune receptor FLAGELLIN SENSING 2 (FLS2) that, upon binding of its ligand, the bacterial PAMP flg22, recruits the co-receptor BRI1-associated receptor kinase 1 (BAK1) to deploy defence signalling. The same co-receptor can also be recruited by other partners for brassinosteroid signalling, and, thus, mediates a cellular decision between defence and growth^8,10^. In addition to protein–protein interactions, lipid heterogeneity can participate in plant immunity^11^. For instance, bacterial lipopeptides^12,13^, or fungal ergosterols^14^, can be recognised by binding to specific lipids or through modulations of lipid-raft structures, activating plant immunity. Also the third functional component, cytoskeleton-membrane interaction, has been detected in the context of defence signalling: the flg22 receptor FLS2 undergoes endocytotic uptake after it has bound its ligand^15^, a process that in plants is intimately linked with the actin cytoskeleton^16^. These three functional components are often acting in concert, as shown for cold and heat stress^17,18^. The membrane stability has been used as a measure of temperature-stress tolerance in plants^19^. Optimal membrane fluidity, one of the fundamental characteristics of biological membranes, determines the stability of the membrane and also affects the adaptation of plants to various stresses. For instance, changes in ambient temperature and osmolarity induce fluctuations in the membrane fluidity^20^. Moreover, for defence, responses of membrane fluidity have been reported: the elicitor cryptogein can activate an increase in membrane fluidity through sterol-binding^21^. Although the membrane fluidity is supported to be a new player in plant defence and several key factors which can influence the status of the membrane fluidity has been identified, such as the steric hindrance and the interactions of its constituents^11^, the mode of action of the membrane fluidity in plant defence remains unclear.

One of the central functions of plant microtubules links intimately with the plasma membrane. They serve as guiding tracks for the movement of cellulose-synthesising complexes within the plasma membrane, and it was actually this membrane-related function responsible for growth axiality that led more than half a century ago first to the prediction of microtubules by Paul Green (1962)^22^, and one year later to their discovery by Ledbetter and Porter (1963)^23^. It does not come as a surprise, therefore, that microtubules are also associated with defence-related membrane dynamics. For instance, using bimodal fluorescence complementation, the co-receptor BAK1 has been shown to align with cortical microtubules, but only, while being associated with the brassinosteroid receptor BRI1, not while being associated with the flg22 receptor FLS2^10^. Microtubules have also been found to be targets of pathogen effectors: HopZ1a, from a plant pathogenic strain of *Pseudomonas syringae*, disrupts cortical microtubules by acetylation of tubulin^24^. This posttranslational modification is characteristic for stable microtubules. It is being mentioned as well that not only microtubules, but also cortical actin filaments are involved in defence responses^25^. Whenever the actin cytoskeleton is genetically or pharmacologically disrupted, the plant’s susceptibility to pathogen infection increases^26^. Several PAMPs and effectors are found to disrupt the actin cytoskeleton to supress plant defence^27^, such as, HopW1, an effector secreted by *Pseudomonas syringae*, which targets F-actin to disrupt actin filaments in vitro and the actin cytoskeleton in planta^28^. The elicitor harpin treatment not only activates Respiratory burst oxidase homologues (RboHs) leading to the accumulation of superoxide anions in the apoplast, but also induces the cellular actin remodelling^29^. Although the interaction between harpin and RboH needs to be further addressed experimentally, it is clear that the diffusion of superoxide anion can activate the glutathionylation of actin^30^. However, it is not clear so far whether the harpin-induced actin reorganisation is caused by the superoxide anions generated by RboH. For other PAMPs (flg22, elf26, and chitin), the knockout of the accompanying receptors (FLS2, EFR, and LYK1) resulting in failure to disrupt the actin organisation^31–33^. Therefore, both microtubules and actin filaments might involve in the signalling transduction of plant defence.

When microtubules are the target of pathogen effectors, they must play a role in defence. This role is, usually attributed to the formation of cell-wall reorganisation such as callosic plugs at the sites of pathogen penetration^34^. When the responses and roles of microtubules were scrutinised, the situation turned out to be more complex. Although in many cases, cortical microtubule arrays were disassembled in response to pathogen attack, the role of this remodelling was found to be discrepant—in some cases supporting pathogen invasion, in others promoting successful defence against the pathogen^35^. These discrepancies point to additional functions of microtubules. In fact, in addition to their well-studied architectural function, microtubules have emerged as important elements of stress perception and transduction^36^. Using cold signalling as a case study, this role of microtubules has been suggested to be that of a susceptor, a structure that translates physical input (in this case, cold-induced membrane rigidification) into a chemical output (in this case calcium influx or apoplastic oxidative burst). This susceptor consists of a functional subpopulation of cortical microtubules acting in concert with the plasma membrane^37^. There are indications that also in defence, microtubules may convey a similar, sensory function: pharmacological compounds acting on microtubules were found to induce defence genes in grapevine cells^38^. Likewise, cold and defence signalling might share their dependence on membrane fluidity: the apoplastic burst triggered by the elicitor cryptogein may modulate plasma membrane fluidity by trapping sterols from the membrane^21^. However, so far, to the best of our knowledge, the interaction of both factors, microtubules and membrane fluidity, has remained unattended.

To characterise the sensory roles of plasma membrane and microtubule network in early defence signalling, we used two transgenic grapevine cell lines that express fluorescent markers for microtubules and actin filaments, respectively. We, then challenged these cells with either flg22, a bacterial elicitor triggering PTI, or with harpin, a bacterial elicitor triggering an ETI-like cell-death-related form of defence, and dissected the resulting cellular signatures, including calcium influx, cytoskeletal responses, expression of defence-related genes, and cell mortality, while modulating membrane fluidity and microtubule network by respective chemical agents. We show that the modulation of plasma membrane or microtubules not only can trigger events of grapevine basal defence, but also modulates the defence responses activated by flg22 and harpin. We find, in addition, that the regulatory effect of membrane rigidification on grapevine defence is independent of both NADPH oxidase and calcium influx, but may relate to the antagonistic immunity triggered by harpin and flg22 in grapevine.

## Results

### Modulation of membrane fluidity can mitigate harpin-triggered elimination of microtubules

The harpin protein from the plant pathogenic bacterium *Erwinia amylovora* is inducing cell-death-related defence responses in the *V. rupestris* cell line. One of these cellular signatures is a depolymerisation of cortical microtubules^7^. Since many cortical microtubules are tethered to the cell membrane^39^, modulations of membrane fluidity might alter the microtubule network and, thus, the microtubular response to harpin.

**Fig. 1:**
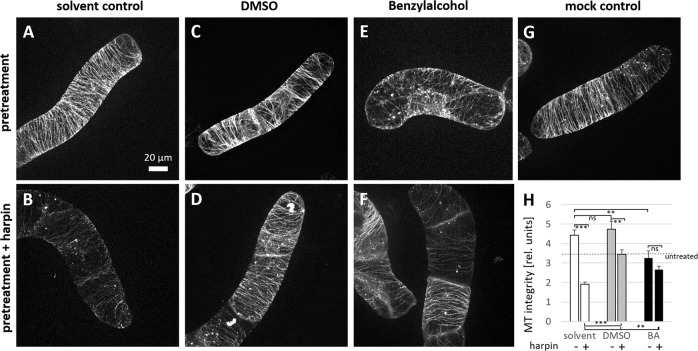
Modulation of membrane fluidity impairs harpin-induced microtubule degradation in *V. rupestris* cells expressing the *GFP-AtTUB6* marker. Geometric projections from z-stacks collected from representative cells imaged by spinning-disc confocal microscopy are shown after pre-treatment with 2% DMSO (**C**), 10 mM BA (**E**), 0.1% DMSO as solvent control (**A**), and water as mock control (**G**) for 30 min, respectively. Then, the cells were treated with 9 μg/ml harpin (**B**, **D**, **F**) for 1 h. Quantitative analysis of microtubule integrity (**H**). Data represent mean and standard error from at least four independent experimental series with 12 to 20 individual cells for each treatment. Significant differences (tested by a Student’s *t* test) are indicated by *P < 0.05; **P < 0.01; ***P < 0.001

To test this, we pre-treated *V. rupestris* cells labelled by the fluorescent tubulin marker *GFP-AtTUB6* for 30 min with DMSO (2% v/v), decreasing fluidity, or with BA, increasing fluidity, prior to treatment with harpin (9 µg mL^−1^) for an additional hour (Fig. 1). Compared to untreated cells (Fig. 1G), the solvent control, treated with 0.1% DMSO, showed denser arrays of transverse microtubules (Fig. 1A). This difference was statistically significant upon quantification of microtubule integrity (Fig. 1H). Nevertheless, these dense microtubule arrays were almost eliminated after treatment with harpin (Fig. 1B), also reflected in a drop of microtubule integrity to around half of the value seen for the solvent alone (Fig. 1H). Pre-treatment with 2% DMSO did produce a dense microtubule array as well (Fig. 1C), albeit there was no significant increase of calculated integrity against the solvent control (Fig. 1H). However, these microtubules were significantly more persistent against harpin treatment (Fig. 1D) as compared to those treated with 0.1% DMSO (Fig. 1B). This difference turned out to be highly significant in the quantification of integrity (Fig. 1H). After pre-treatment with 10 mM BA (Fig. 1E), microtubules were seen in partially depleted arrays of thinner and also less ordered microtubules as compared to the solvent control, although there was no significant difference in terms of integrity, if compared to untreated cells (Fig. 1H). Although these microtubules in some cells appeared thinner and replaced by punctate signals in response to harpin, not all cells showed this phenomenon cells (Fig. 1F). In the quantification, the overall effect turned out to be minor and not significant (Fig. 1H), such that the harpin treatment affected microtubules significantly less compared to the pre-treatment with 0.1% DMSO. Thus, harpin eliminates microtubules in a stringent manner, which can be largely suppressed by 2% DMSO, but also by benzyl alcohol, although the pre-treatment causes different levels of microtubule bundling (high for 2% DMSO, absent for benzyl alcohol).

### Taxol renders microtubules persistent to harpin

**Fig. 2:**
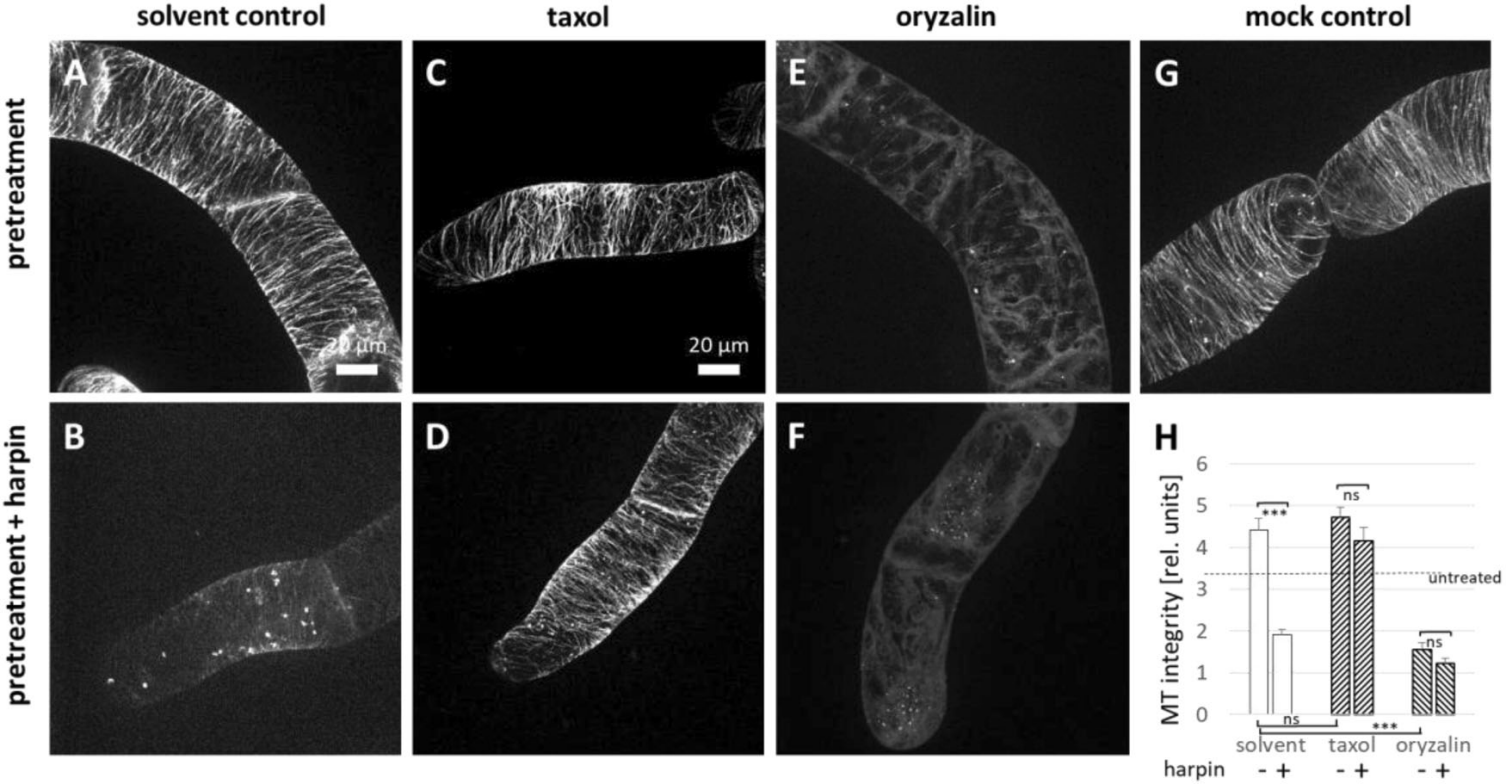
Modulation of microtubules alters harpin-induced microtubule degradation in *V. rupestris* cells expressing the *GFP-AtTUB6* marker. Geometric projections from z-stacks collected from representative cells imaged by spinning-disc confocal microscopy are shown after pre-treatment with 10 μM taxol (**C**), 10 μM oryzalin (**E**), 0.1% DMSO as solvent control (**A**), and water as mock control (**G**) for 30 min, respectively. Then, the cells were treated with 9 μg/ml harpin (**B**, **D**, **F**) for 1 h. Quantitative analysis of microtubule integrity (**H**). Data represent mean and standard error (SE) of the mean from at least four independent experimental series with 12 to 20 individual cells for each treatment. Significant differences (tested by a Student’s *t* test) are indicated by *P < 0.05; **P < 0.01; ***P < 0.001

Since above works have verified that the membrane fluidity changes can induce clear microtubule reorganisation and mitigate harpin-triggered elimination of microtubules (Fig. 1), it would be necessary to check the direct effects of microtubule drugs (taxol and oryzalin) on the harpin-elicited disruption. In the next step, we tested the effect of direct pharmacological manipulation of microtubules on harpin-induced elimination (Fig. 2). Pre-treatment with 10 µM taxol for 30 min (Fig. 2C) caused a slight bundling and a reduced number of microtubules as compared to the solvent control with 0.1% DMSO (Fig. 2A) or mock-treated cells (Fig. 2G). These bundled microtubules were found to persist a subsequent treatment with harpin over 1 h (Fig. 2D). In contrast, a pre-treatment with 10 µM oryzalin over 30 min efficiently eliminated microtubules (Fig. 2E) and, as to be expected, the microtubules remained absent also during the subsequent treatment with harpin (Fig. 2F). A quantification over microtubule length (Fig. 2H) confirmed that the taxol pre-treatment stabilised microtubules in a similar manner as did a membrane rigidifying treatment with 2% DMSO (Fig. 1H).

### Modulation of membrane fluidity can mitigate harpin-triggered elimination of actin filaments

**Fig. 3:**
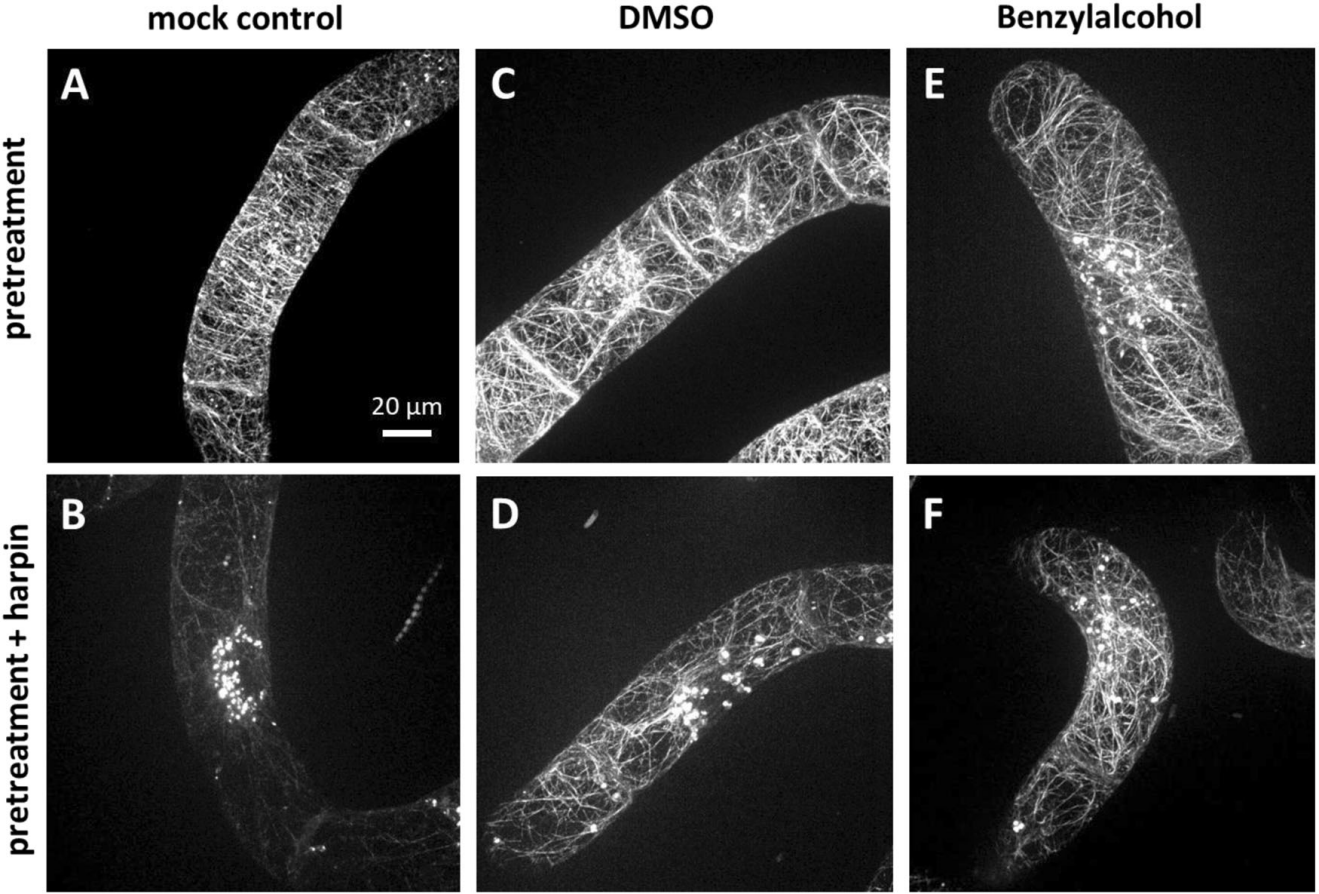
Modulation of membrane fluidity impairs harpin-induced actin filaments disruption in *V. vinifera* cells expressing the *GFP-FABD2* marker. Geometric projections from z-stacks collected from representative cells imaged by spinning-disc confocal microscopy are shown after pre-treatment with 2% DMSO (**C**), 10 mM BA (**E**), and water as mock control (**A**) for 30 min, respectively. Then, the cells were treated with 9 μg/ml harpin (**B**, **D**, **F**) for 1 h

We asked next, whether only microtubules respond to modulators of membrane fluidity. To probe the behaviour of actin filaments in grapevine cells, a FABD2-GFP marker strain generated in *V. vinifera* cv. ‘Chardonnay’ was pre-treated with DMSO or BA (along with an untreated control) for half an hour, and then incubated with harpin for 1 h. In the untreated control, the fine meshwork of cortical actin filaments (Fig. 3A) was almost completely eliminated in response to harpin, while agglomerations of punctate signals accumulated around the nucleus, along with faint trans-vacuolar cables (Fig. 3B). In response to 2% DMSO, the actin filaments were mildly bundled and the trans-vacuolar cables became more prominent, with occasional actin dots close to the nucleus (Fig. 3C). Subsequent treatment with harpin affected this actin organisation to a certain extent, evident from the appearance of the perinuclear actin dots (Fig. 3D). However, compared to the untreated control (Fig. 3B), the actin cytoskeleton was only mildly affected and, thus, was more persistent to harpin treatment. Pre-treatment with BA yielded a similar pattern (Fig. 3E) to that seen for DMSO pre-treatment (Fig. 3C), such as mild bundling of cortical filaments, the appearance of trans-vacuolar actin cables, and a few perinuclear actin dots. In addition, the pattern produced by subsequent harpin treatment was very similar to that seen after DMSO pre-treatment (Fig. 3D), i.e. the actin cytoskeleton persisted to harpin (Fig. 3F). Thus, both DMSO, a compound that renders the membrane more rigid, and BA, a compound that renders the membrane more fluid, were stabilising the actin cytoskeleton against the effect of harpin. This means that not only microtubules but also actin filaments respond in a paradox manner.

### Modulation of membrane fluidity can activate defence responses

**Fig. 4:**
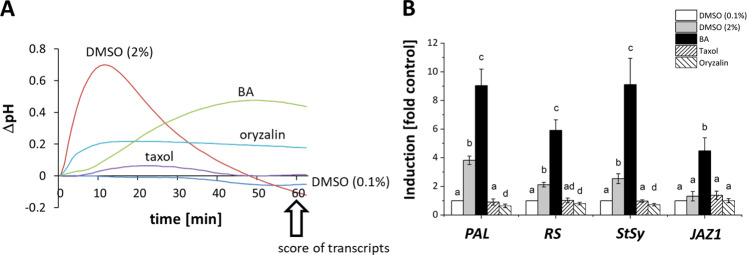
Defence responses to modulation of membrane fluidity and microtubules in *V. rupestris* cells expressing the *GFP-AtTuB6* marker. **A** Extracellular alkalinisation in response to 2% DMSO, 10 mM BA, 10 μM taxol, 10 μM oryzalin, and 0.1% DMSO as solvent control. **B** Steady-state transcript levels for defence genes quantified by qRT-PCR in response to the same compounds administered for 1 h. The phytoalexin synthesis genes *Phenylalanine Ammonia Lyase 1* (*PAL*); *Resveratrol Synthase* (*RS*); *Stilbene Synthase* (*StSy)*; and the jasmonate response marker *Jasmonate ZIM/TIFY-Domain Protein 1* (*JAZ1*) were measured against *Elongation Factor 1-α* (*EF1-α*) as internal standard for quantification. Data represent mean and standard error from three biological replicates with three technical replicates each. Means denoted by different letters are significantly different at *P* < 0.05 by a Student’s *t*-test

Since modulation of membrane fluidity can mitigate the response of both microtubules (Fig. 1) and actin filaments (Fig. 3), to the bacterial elicitor harpin, we asked further, whether membrane fluidity is also relevant for defence responses. Calcium influx has been reported as one of the earliest cellular responses in plant defence^40,41^. This calcium influx can be easily recorded by measuring the extracellular alkalinisation^42^. To test, whether modulations of membrane fluidity or microtubules would induce this early readout of defence, we measured extracellular alkalinisation triggered by DMSO, BA, taxol, and oryzalin in *V. rupestris* GFP-TuB6 cells. Both, DMSO and BA, clearly activated calcium influx, but with a different time course (Fig. 4A). In response to 2% DMSO, the pH increased immediately and very rapidly to a maximum of 0.7 units reached within around 11 min. Subsequently, pH gradually returned to the initial level over an interval around 40 min. A pre-treatment with GdCl_3_, an inhibitor of calcium influx suppressed this increase of pH (Supplementary Fig. S1A), which is evidence for the hypothesis that the change of pH is due to calcium influx. Likewise, BA induced an alkalinisation with a peak of around 0.5 units. However, this response showed a lag phase of almost 10 min and developed then slowly reaching a peak only as late as around 50 min after onset of the treatment. Moreover, GdCl_3_ failed to suppress the pH response induced by BA (Supplementary Fig. S1B). Thus, BA seems to act by different mechanisms. Oryzalin induced a mild and significant response of pH with 0.18 units, while taxol failed to evoke any significant response (Fig. 4A). The results indicated that membrane rigidification by DMSO directly activates a fast calcium influx, while increased membrane fluidity by BA does not. The elimination of microtubules by oryzalin is able to deploy calcium influx, but the stabilisation of microtubules by taxol not.

In the next step, we asked, whether the activation of calcium influx would lead to the induction of defence-related transcripts in this cell line (*V. rupestris* GFP-TuB6). We probed for genes involved in phytoalexin synthesis, such as phenylammonium lyase (*PAL*) as first committed step of the phenylpropanoid pathway, as well as stilbene synthases (*StSy*) and resveratrol synthase (*RS*) as key enzymes of stilbenoid synthesis that are rapidly induced during PTI^7^. To probe for the activation of PTI, we used the jasmonate response factor JAZ1^43^ as a marker. When we measured steady state transcript levels in response to modulation of membrane fluidity, or microtubules, respectively, (Fig. 4B), we observed that BA caused clearly induced all tested transcripts (*PAL* 9-fold, *RS* 6-fold, *StSy* 9-fold, *JAZ1* 5-fold). In contrast, DMSO activated the phytoalexin-synthesis transcripts, albeit to a weaker extent (*PAL* 4-fold, *RS* 2-fold *StSy* 3-fold), but not *JAZ1*. Thus, the pattern seen on the level of defence-related transcripts did not reflect that for extracellular alkalinisation. DMSO that triggered a strong and rapid pH response (Fig. 4A) induced transcripts more weakly (Fig. 4B) as compared to BA, which only had produced a sluggish pH response, which did not depend on calcium channels (Supplementary Fig. S1B). In contrast to the membrane fluidity modulators DMSO and BA, the microtubule-targeted compounds taxol and oryzalin did not produce any significant change in transcript levels (Fig. 4B).

### Modulation of membrane fluidity can silence harpin-triggered gene expression, but not extracellular alkalinisation

**Fig. 5:**
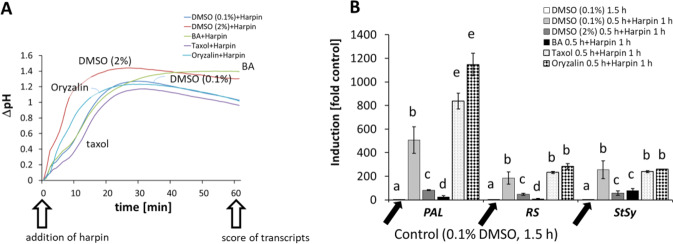
Defence responses to bacterial elicitor harpin after modification of membrane fluidity and microtubules in *V. rupestris* cells expressing the *GFP-AtTuB6* marker. The concentration of harpin was 9 μg/ml, treatment time was 1 h. Extracellular alkalinisation (**A**) and steady-state transcript levels of the phytoalexin-synthesis genes *PAL*, *RS*, and *StSy* (**B**) were monitored against Elongation Factor 1*-α* (EF1*-α*) as internal standard for quantification. Data represent means and standard error from three biological replicates with three technical replicates each. Means denoted by different letters are significantly different at *P* < 0.05

Since DMSO and harpin had been found to cause both, extracellular alkalinisation (Fig. 4A) and activation of defence-related genes (Fig. 4B), we wondered, whether these compounds would also modulate defence responses triggered by the bacterial elicitor harpin^7^. To test this, we pre-treated the *V. rupestris* GFP-TuB6 cells for 30 min with either DMSO (2%) or BA prior to activating defence by addition of harpin. In a parallel set of experiments, we assessed the effects of taxol and oryzalin, along with a solvent control (0.1% DMSO). The defence response was monitored either at the level of extracellular alkalinisation (Fig. 5A), or at the level of phytoalexin-synthesis transcripts (Fig. 5B). The membrane rigidifier DMSO (2%) amplified the extracellular alkalinisation induced by harpin by around 0.2 pH units (Fig. 5A). This amplification initiated immediately after addition of harpin, and persisted subsequently. In contrast, BA did not modulate the alkalinisation in response to harpin over the initial 30 min. However, BA efficiently enhanced the pH responses after 30 min (Fig. 5A). Taxol and oryzalin could not modulate the alkalinisation stimulated by harpin (Fig. 5A). These data suggested that membrane rigidification promotes harpin-activated calcium influx, while the status of microtubule network, whether bundled or depolymerised, had no effect.

To verify the direct links between extracellular alkalinisation and calcium influx induced by various chemical treatments, the cytosolic calcium under different treatments was labelled by chloro-tetracycline according to Doniak et al.^44^. Compared to the solvent (water) control, the fluorescence was significantly increased in cells after treating with different chemicals for 25 min. The differences could be clearly observed and distinguished between the representative example DMSO-treated cell and water-treated cell (Supplementary Fig. S2A). Moreover, the green signal distribution was displayed in a quantitative manner via measuring the change of skewness on cells treated with various chemicals^45^. The results revealed that all treatments have induced significant increase in the skewness, which were consistent with the image observations (Supplementary Fig. S2B). These findings indicated that the various chemicals can induce clear and significant calcium influx. In agreement, these chemicals also elicited significant extracellular alkalinisation in our study (Figs. 4A and 5A).

In the next step, we probed for modulations of harpin-induced gene expression (Fig. 5B). Compared to the solvent control (1.5 h 0.1% DMSO), harpin by itself induced a massive induction of phytoalexins-transcripts, ranging from around 200-fold (for *RS*) till 500-fold (for *PAL*). Both, rigidification (by 2% DMSO) and fluidisation (10 mM BA) suppressed this induction by harpin almost completely, which is in stark contrast to the relatively mild modulation of extracellular alkalinisation (Fig. 5A). For 2% DMSO, the effects on pH (enhancement) and gene expression (strong suppression) are even antagonistic. For *RS* and *StSy*, there was no effect, neither taxol, nor oryzalin, which was in line with the lacking effect of these compounds on extracellular alkalinisation. The pattern for *PAL* was different. Here, the already conspicuous induction by harpin (around 500-fold) was amplified further to more than 800-fold (for taxol), and almost 1200-fold (for oryzalin).

Thus, the early defence responses can be uncoupled from the induction of phytoalexins genes. Any change of membrane fluidity, whether it may be an increase or a decrease can suppress the harpin response of gene expression. Modulation of microtubules enhances *PAL*, but not the other tested phytoalexin-related transcripts. Again, this effect holds true for both, an increase as well as for a decrease of microtubule stability.

### Modulation of membrane fluidity can boost flg22-triggered gene expression

**Fig. 6:**
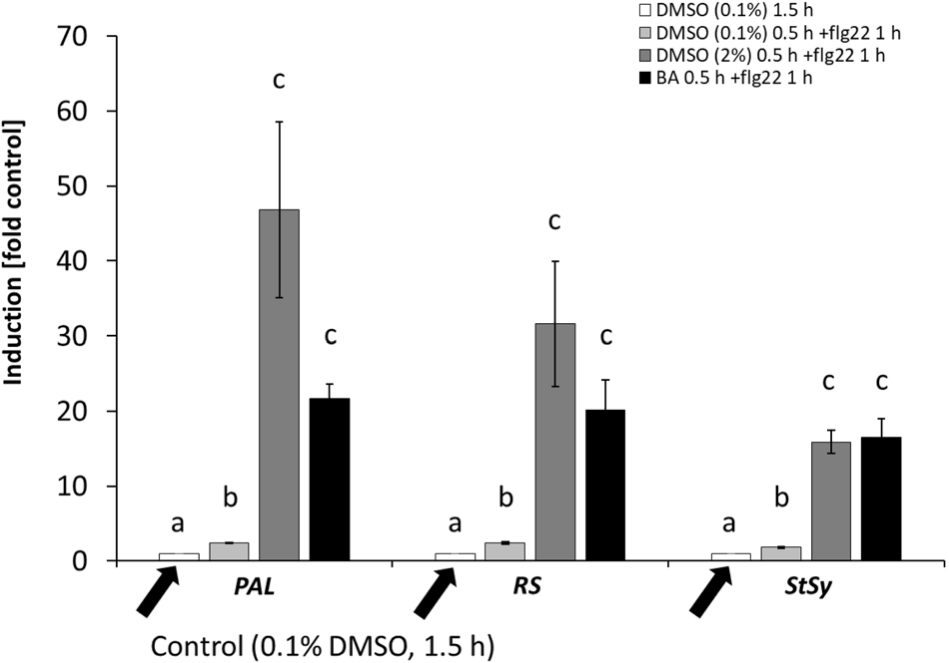
Modulation of membrane fluidity alters flg22-triggered genes expression in *V. rupestris* cells expressing the *GFP*-AtTuB6 marker. Steady-state transcript levels for the phytoalexin synthesis genes *PAL*, *RS*, and *StSy* were measured after 1 h treatment with 1 µM of the bacterial elicitor flg22 or 0.1% DMSO as solvent control, following pre-treatment with either 2% DMSO or 10 mM BA, or with 0.1% DMSO for half an hour. Elongation Factor 1*-α* (EF1*-α*) served as internal standard for quantification. Data represent means and standard error from three biological replicates with three technical replicates each. Means denoted by different letters are significantly different at *P* < 0.05

In the next step, we investigated, whether the effect of membrane fluidity on the defence response of phytoalexin transcripts was dependent on the type of defence. For this purpose, we triggered PTI as alternative response by the bacterial elicitor flg22 in *V. rupestris* GFP-TuB6 cells. Before, we pre-treated the cells with either 0.1% DMSO (as solvent control), with 2% DMSO (as rigidifier), or 10 mM BA (as fluidiser) for half an hour (Fig. 6). While flg22 alone caused a mild (2-fold), but significant induction for PAL, RS, and StSy, 2% DMSO and 10 mM BA strongly boosted this response. In the case of *PAL*, DMSO amplified the response around 20-fold, and BA around 10-fold. For *RS*, the amplification by DMSO was less (around 15-fold versus 10-fold for BA), for *StSy* both compounds amplified around 10-fold. Overall, the effect of fluidity modulation on flg22-triggered gene expression was just opposite to that seen for harpin-triggered gene expression.

### Silencing of the harpin response by DMSO does not depend on calcium influx

**Fig. 7:**
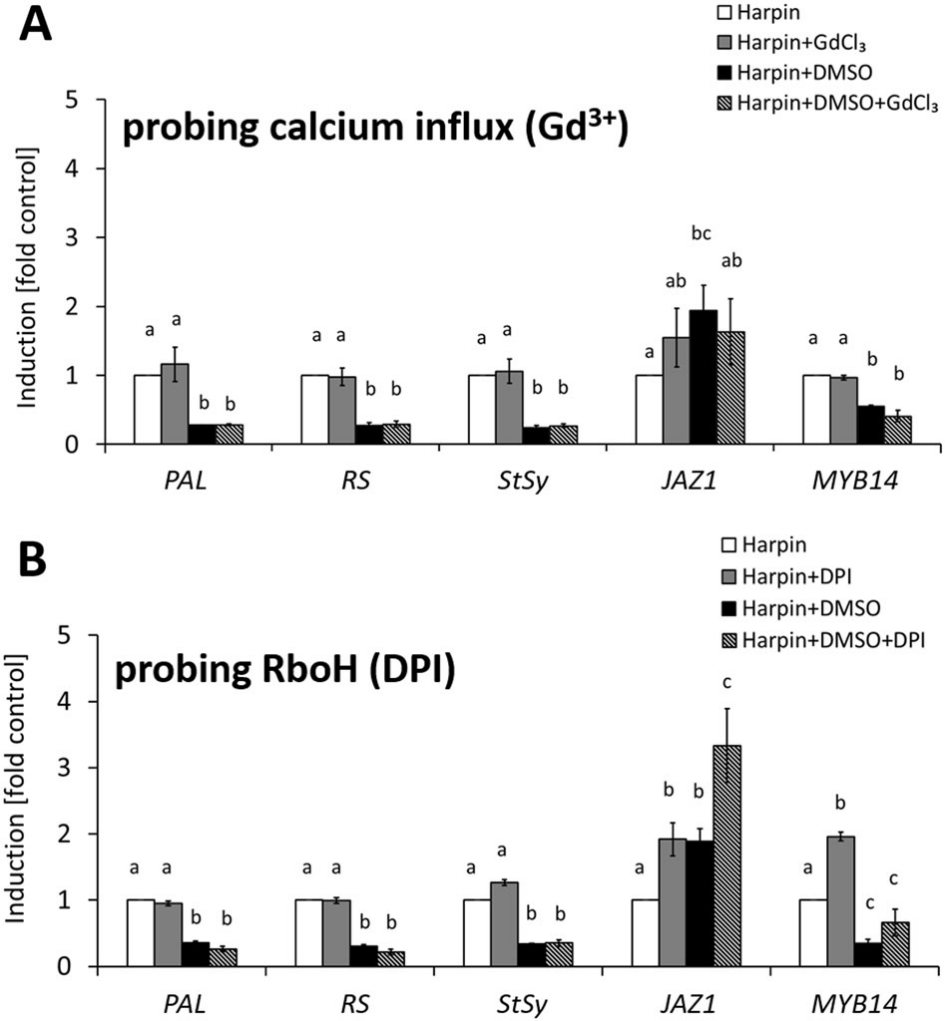
Role of Ca^2+^ and ROS on DMSO-modulated harpin-induced genes expression. The calcium-channel blocker gadolinium chloride (GdCl_3_, 100 µM, **A**) and the RboH blocker Di-Phenylene Iodonium chloride (DPI, 10 µM, **B**), were administered together with the bacterial elicitor harpin for one hour. Then, steady-state transcript levels for the phytoalexin-synthesis genes *PAL*, *RS*, and *StSy*, the jasmonate response regulator *JAZ1*, and *MYB14* (a transcription factor activating stilbene synthase genes) were measured against Elongation Factor 1*-α* (EF1*-α*) as internal standard for quantification. Data represent means and standard error from three biological replicates with three technical replicates each. Means denoted by different letters are significantly different at *P* < 0.05

Since membrane rigidification was found to induce significant calcium influx (Fig. 4A) that can be blocked by GdCl_3_ (Supplementary Fig. S1A), while also activating defence-related transcripts (Fig. 4B), we wondered, whether also this activation of transcripts was dependent on calcium influx. To test this, we disrupted calcium influx by GdCl_3_ before testing gene expression in response to a combined treatment with DMSO and harpin. In contrast to the previous experiment, where we had added DMSO 30 min prior to harpin, we administered the compounds simultaneously, to extract also information on the time course of their action. In addition to the genes tested previously, *MYB14* was included, a transcription factor activating the stilbene synthase promoter^46^. Similarly to the experiments, where DMSO had been added prior to induction of defence by harpin (Fig. 5B), the induction of *PAL*, *RS*, and *StSy* by harpin was suppressed, indicating that the effect of DMSO was instantaneous (Fig. 7A). Likewise, *MYB14* was suppressed, while *JAZ1* was not. For none of the four tested transcripts, where DMSO exerted this inhibitory effect, did the addition of GdCl_3_ to DMSO cause any significant change compared to DMSO alone. Also for *JAZ1*, where DMSO was not inhibiting the induction by harpin, GdCl_3_ as third component did not make any difference (Fig. 7A).

To address whether Ca^2+^ might play a role for harpin-induced gene expression in presence of BA, we also conducted a parallel experiment by replacing DMSO by BA. However, there were no significant differences between the transcript levels, no matter, whether we administered harpin alone, or in combination with BA, or with both, BA and GdCl_3_ in combination (Supplementary Fig. S3). Thus, consistently with the lacking effect of GdCl_3_ on BA-induced alkalinisation (Supplementary Fig. S1), the status of calcium influx neither impaired harpin-induced gene expression, nor show any interaction with BA in this respect.

### DMSO silencing depends on RboH for regulating, not for metabolic, genes

In addition to calcium influx, a group of NADPH oxidases located in the plasma membrane, the Respiratory burst oxidase homologues (RboHs), are central for stress signalling^47^. Diphenylene iodonium (DPI), a specific inhibitor of RboH, can suppress harpin-induced gene expression in grapevine^48^. We asked, therefore, whether DPI would suppress the silencing effect of DMSO upon harpin-induced gene expression.

The experiment was following the same design as described above, just replacing GdCl_3_ by DPI. The inhibitor DPI alone did not affect the induction of *PAL*, *RS*, and *StSy* by harpin, nor did it mitigate the suppression of this induction by DMSO (Fig. 7B). In contrast, DPI alone already enhanced the expression of *JAZ1* and *MYB14* as components involved in basal immunity to around two fold. In combination with DMSO, DPI amplified the induction of *JAZ1* by harpin. Thus, the response of defence-related genes bifurcates into two patterns. While the induction of phytoalexins synthesis genes (*PAL, RS, StSy*) by harpin does not show any dependency on DPI, neither if given alone nor in combination with DMSO, the harpin response of the immunity signalling genes *JAZ1* and *MYB14* is already elevated by DPI alone, and in case of *MYB14* is even further accentuated by DMSO.

### Modulation of membrane fluidity amplifies harpin-induced cell mortality

**Fig. 8:**
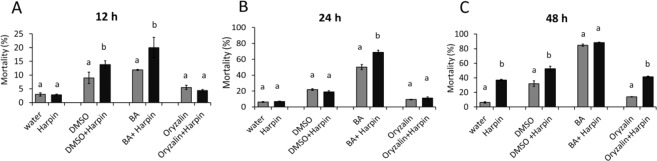
Effect of modulating membrane fluidity and microtubules on the harpin-induced cell mortality in *V. rupestris* cells expressing the *GFP-TuB6* marker. The cells were incubated with 2% DMSO, 10 mM BA, and 10 μM oryzalin alone, or combined with 9 μg/ml harpin for 12 h (**A**), 24 h (**B**), and 48 h (**C**). Treatment with 9 μg/ml harpin alone served as positive control, treatment with water as solvent control. Cell mortality was scored by the Evans Blue Dye Exclusion Assay. Data represent means and standard error. Means denoted by different letters are significantly different at *P* < 0.05

To probe for a potential role of membrane fluidity and microtubules in defence-related cell mortality, we followed mortality over time in *V. rupestris* TuB6 cells in response to either water, DMSO, BA, or oryzalin alone or in combination with harpin, respectively. While oryzalin did not cause a significant increase in mortality, both DMSO and BA progressively raised mortality over time, most pronounced for BA. For instance, after 24 h, the cell mortality was around 6.1% in the water control, but had increased to 50% in the BA treated sample (Fig. 8B). Moreover, if given in combination with harpin, both, DMSO and harpin enhanced mortality beyond the value seen for harpin alone (Fig. 8C). However, oryzalin failed to induce or modulate grapevine cell death (Fig. 8A–C). Thus, DMSO, and especially BA, can activate mortality by themselves and enhance the harpin-induced cell death. In contrast, microtubules do not seem to play a role here.

## Discussion

**Fig. 9:**
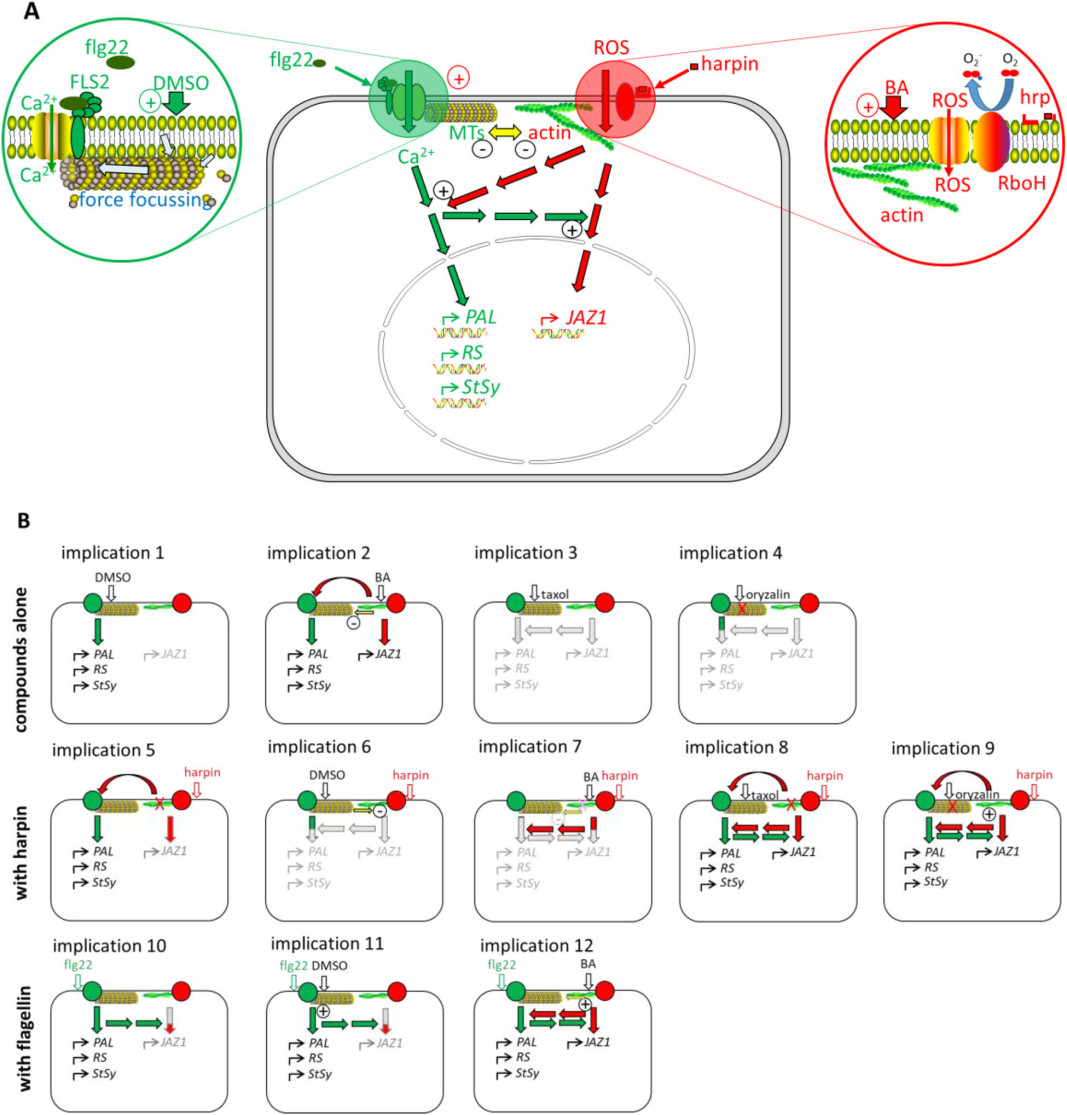
Working model (A) and implications (B) for membrane-associated immune responses in grapevine. **A** The working model for defence triggered by flg22 (green) and harpin (red): flg22 is perceived by the membrane receptor FLS2, causing a rapid calcium influx, which partially activates transcription of genes *PAL*, *RS*, *StSy* or gene *JAZ1*. DMSO decreases plasma membrane fluidity, causes microtubule reorganisation, which also induces calcium influx. Harpin activates RboH, changing actin, which will destabilise microtubules, this activates phytoalexins synthesis genes and *JAZ1*. Apoplastic ROS from RboH can secondarily activate the calcium channel, leading to a delayed pH response. BA increases plasma membrane fluidity, modulating RboH activity. **B** Implications derived from the working model shown in **A**: **Implications 1–4** Events predicted by the model for incubation with modulators of membrane fluidity (DMSO, BA), or microtubules (taxol, oryzalin); **Implications 5–9** Events predicted by the model for treatment with harpin alone or in combination with modulators or membrane fluidity or microtubules; **Implications 10–12** Events predicted by the model for treatment with flg22 alone or in combination with modulators or membrane fluidity or microtubules

In addition to serving as a selective barrier, the plant plasma membrane plays a sensory role in perceiving and translating external signals into appropriate innate responses^49^. This is not confined to chemical signalling based on binding of ligands to receptors in the plasma membrane, but also extends to physical signallings, such as high temperature^50^, cold stress^51^, or mechanic stimuli, such as osmotic tension^52^, touch^53^, or gravity^54^. How these physical signals translate into downstream chemical signals has remained enigmatic, though. Microtubules are quite rigid structures—their Young modulus is comparable to that of glass^55^—and interact closely with the membrane. They might serve to amplify the minute forces originating from signal-dependent changes of membrane fluidity. The role of this microtubule-membrane fluidity circuit for cold sensing has been recently reviewed^37^. The finding that membrane-fluidity changes are also a component of early defence responses^21^ motivated our question, whether the microtubule-membrane fluidity circuit can deploy defence as well. We addressed this in grapevine cells expressing a GFP-tagged tubulin, such that we could follow the responses of microtubules to modulations of membrane fluidity. We find that DMSO, a membrane rigidifier, can cause microtubular bundling, accompanied by defence responses, including activation of ion channels, and expression of phytoalexins genes. On the other hand, DMSO can suppress the gene expression in response to the bacterial elicitor harpin, while amplifying the response to the bacterial elicitor flg22. We also find that BA, although being a membrane fluidiser, can evoke partially overlapping effects to DMSO. The following discussion will explain these specific and complex patterns by a unifying model (Fig. 9A), where the microtubule-membrane fluidity circuit interacts differentially with two major inputs for defence signalling, i.e. calcium influx and apoplastic oxidative burst caused by the membrane located NADPH oxidase Respiratory burst oxidase Homologue (RboH)^7^. In the following, we spell out different implications of this model (Fig. 9B) and compare it to experimental evidence.

### Membrane rigidification and fluidisation activate different aspects of defence

That DMSO and BA are both able to activate defence, although their effect on membrane fluidity is opposite, seems paradox. We explain this by the activation of different signal pathways that are partially antagonistic, but partially act in synergy. Our model derives from the concept, where the mechanical forces originating from DMSO-mediated membrane rigidification can induce basal immunity through the activation of calcium influx involving microtubules. When the membrane fluidity drops in specific patches, a minute force arises along the borderline of fluid and less fluid patches^37^. Cortical microtubules (or a specific subset of these) integrate these forces into a net force by virtue of their high rigidity and their ability to transmit vibrations^56^. The calcium influx deploys then a signal moving to the nucleus, probably involving a MAPK cascade, culminating in the activation of phytoalexin-synthesis genes. This aspect of our model can explain the activation of extracellular alkalinisation by DMSO (Figs. 4A and 9B, *implication 1*), the stabilisation of microtubules (Figs. 1C and 9B, *implication 1*) as well as the induction of phytoalexin synthesis genes (Fig. 4B, *implication 1*), and the strong stimulation of flg22 induced gene activation (Figs. 6 and 9B, *implication 10*). Stabilisation of microtubules alone (by taxol) is not sufficient to activate these responses (Fig. 4). A similar phenomenon occurs in cold acclimation, where microtubule stabilisation alone does not induce signalling, while it can promote hardiness in combination with cold stress^18^. Microtubules are, therefore, not part of sensing itself. However, they amplify the responsiveness of the sensor^37^.

Membrane fluidisation by BA acts obviously different from DMSO, because it is stabilising microtubules, if acting alone (Fig. 1E), but can stabilise them against harpin-induced elimination (Fig. 1F). Since gene activation by harpin can be disrupted by diphenylene iodonium^48^, and since this process depends on actin remodelling^29^, we assign the inducing effect of BA to the activation of the NADPH oxidase Respiratory burst oxidase Homologue (RboH) as the primary input. Since reactive oxygen species in the apoplast are able to activate calcium influx^57^, BA is expected to induce apoplastic alkalinisation similar to DMSO. However, since the activation is indirect, this activation should proceed with a slower time course. This implication of our model (Fig. 9B, *implication 2*) is exactly, what we observe (Fig. 4A). A further indication for separate pathways is the fact that BA, in contrast to DMSO, can induce *JAZ1* transcripts (Fig. 4B). The different timing of calcium influx may mean that, for DMSO, calcium influx acts as early and primary signal, while for BA calcium influx is a consequence, rather than the cause of primary signalling. This model of two signalling inputs integrate smoothly with the finding that cold and DMSO (i.e. factors leading to membrane rigidification) activate a MAPK cascade differing from that deployed by heat and BA (i.e. factors leading to membrane fluidisation) meaning that the bifurcation in signalling must be upstream of MAPK signalling^17^. Since heat-induced membrane-fluidisation activates RboH in tobacco cells^50^, the physical effect of BA (membrane fluidisation) should activate RboH as well. Furthermore, BA did not activate the expression of the cold adaptation factor *CBF4*, which is strictly dependent on calcium influx^18^. This means that BA must activate an input different from calcium influx, and this input is most likely RboH, consistent with *implication 2* of our model (Fig. 9B).

### The bacterial elicitors, flg22 and harpin, activate different aspects of defence

A second paradox is the different effect of membrane rigidification on the response to flg22 and harpin. This effect is just the reverse—the transcript response to harpin decreases strongly, the transcript response to flg22 increases strongly. We resolve this paradox by assigning the primary effect of these elicitors to the two different signalling inputs described above: flg22 is primarily targeting the calcium-influx microtubule hub, while harpin is primarily targeting the RboH-actin hub (Fig. 9A). We were able to test and confirm several implications of this model: for instance, rigidification of the membrane by DMSO should amplify extracellular alkalinisation in response to flg22 as well as transcripts of phytoalexin response genes (Fig. 9B, *implication 11*) consistent with the experimental record (Fig. 6). Instead, the activation of extracellular alkalinisation by BA should occur indirectly (Fig. 9B, *implication 2*), such that its time course is delayed, again congruent with our data (Fig. 4A). A similar delay of extracellular alkalinisation compared to the swift activation by flg22 occurs in response to harpin^7^. Membrane rigidification by DMSO should also suppress the eliminating effect of harpin on microtubules (Fig. 9B, *implication 6*) as does a pre-stabilisation through taxol (Fig. 9B, *implication 8*), what we actually observe (Figs. 1D and 2D, respectively). The cytoskeleton acts as an amplifier, not as a transducer on the primary inputs calcium influx and RboH activity (Fig. 9A). As a result, microtubule stabilisation by taxol should not be able to deploy signalling (Fig. 9B, *implication 3*), and microtubule elimination by oryzalin should only cause a minor calcium efflux by removing the gating function of microtubules (Fig. 9B, *implication 4*), which is matching our observations (Fig. 4A, B). Our results are also in line with measurements of cold-induced calcium influx in tobacco^58^. These authors observed as well a mild calcium influx upon treatment with oryzalin, while taxol had no effect. We conclude that modifications of membrane fluidity modulate elicitor perception rather than signal transduction.

### Signalling to phytoalexin synthesis genes and jasmonate signalling genes differs

Phytoalexin synthesis genes of grapevine are active for both, PAMP-triggered immunity in response to flg22 and cell-death related immunity in response to harpin^7^, while the jasmonate-signalling gene *JAZ1* is only responsive to flg22^43^. This bifurcation is also manifest in the current study. We, therefore, place the two groups of transcripts under control of separate, but cross-talking, signalling chains (Fig. 9A). The phytoalexin-synthesis transcripts respond primarily to calcium influx, *JAZ1* primarily to oxidative burst through RboH. Membrane rigidification by DMSO should, through activation of calcium influx, induce all three tested phytoalexin-synthesis transcripts, but fail to do so for *JAZ1* (Fig. 9B, *implication 1*), which is exactly, what we observe (Fig. 4B). Instead, membrane fluidisation by BA should, through triggering RboH, activate *JAZ1* and, through the secondary activation of calcium influx, induce the phytoalexin-synthesis genes as well (Fig. 9B, *implication 2*), which is again confirmed by our experimental data (Fig. 4B). Membrane rigidification by DMSO should suppress the harpin-induced modelling of actin through the stabilisation of microtubules. As a consequence, phytoalexin-synthesis genes should remain silent as well (Fig. 9B, *implication 6*). In fact, DMSO stabilises both, microtubules (Fig. 1D) and actin filaments (Fig. 3D) against elimination by harpin, and quells the induction of the phytoalexin-synthesis genes (Fig. 7). The fact that flg22 can activate *JAZ1*, which is poorly activated by harpin^43^ indicates a positive regulation of *JAZ1* by calcium signalling (Fig. 9B, *implication 10*) while activation of phytoalexin-synthesis genes through BA implies a positive regulation of calcium triggered signalling by RboH (Fig. 9B, *implication 12*). This regulation seems to differ from the activation of calcium influx by apoplastic ROS, because transcripts already are up at a time, where apoplastic alkalinisation is just initiating (Fig. 4A). This cross talk would also imply that BA should amplify the response to flg22 (Fig. 9B, *implication 12*), which is again in line with the experimental evidence (Fig. 6). It is not known, at what level this cross-talk is taking place, but a plausible working hypothesis would locate it to the MAPK signalling cascade, which could be tested in the future using the MAPK inhibitor PD98059^7^.

### Microtubules are not part of signalling, but they modulate signalling

The response of microtubules to the bacterial elicitor was rapid and was suppressed (DMSO) or at least weakened (BA) by modulation of membrane fluidity (Figs. 1 and 2). To understand, whether this response was just a by-product of defence, or whether it was involved in signalling, we used oryzalin (eliminating microtubules) and taxol (stabilising microtubules) before testing defence responses. The outcome was partially paradox, since both compounds were acting in parallel, although their effect on microtubules is antagonistic. For instance, oryzalin was able to amplify the activation of *PAL* transcripts by harpin significantly (Fig. 5B), and pretreatment with taxol yielded the same effect, although taxol was stabilising microtubules against harpin (Fig. 2D). Similarly, both taxol and pronamide (induces a transient elimination of microtubules) treatments could induce the cold hardening to subsequent cold stress^18,37^. Therefore, the microtubules definitely do not transduce the defence or cold signals, but they play a role in defence response or cold hardening. In this study, the other phytoalexin-synthesis transcripts did not show this amplification, which might be linked with the fact that the overexpression of the GFP-tubulin marker mildly reduces microtubule dynamics^59^, such that less sensitive transcripts might not show the amplification. To resolve a paradox, one needs some kind of bifurcation. We incorporated, therefore, a dual role of microtubules into our model (Fig. 9A): on the one hand, they act negatively on RboH dependent signalling, possibly by interacting with actin filaments^60^, on the other, they participate in the regulation of calcium influx. Upon elimination of microtubules by oryzalin, the gating of calcium channels ceases (Fig. 9B, *implication 4*), which, in absence of a primary membrane fluidity response, does not lead to gene activation. In fact, oryzalin treatment deploys a mild, but swift extracellular alkalinisation (Fig. 4A), which is not accompanied by a significant activation of defence-related transcripts, at least not of those tested in this study (Fig. 4B). In combination with harpin, oryzalin should boost the harpin response, because it removes the negative impact of microtubules on RboH dependent signalling (Fig. 9B, *implication 9*). Taxol treatment would not deploy calcium influx in the absence of a membrane fluidity response (Fig. 9B, *implication 3*). Again, this implication is in congruence with the experimental data (Fig. 4A, B). In combination with harpin, the stabilised microtubules would amplify RboH dependent signalling, probably by impairing actin filaments (Fig. 9B, *implication 8*) and, thus, amplify the gene response to harpin, which is what we observe (Fig. 5B). This amplification should occur without an increase in calcium influx. Consistent with this implication, we do not see any change of extracellular alkalinisation in response to harpin by taxol (Fig. 5A). Therefore, we concluded that microtubule is certainly not a transducer but an amplifier of signals.

It is very unlikely that microtubules act as part of signalling, because neither treatment with oryzalin, nor with taxol could induce significant gene activation (different from the outcome seen for a modulation of membrane fluidity (Fig. 4)). Microtubules rather act as modulators of the signalling deployed by changes of membrane fluidity. This shifts the focus on functional subdivision of the membrane into so-called nano-domains^49,61^. For instance, microtubules can modulate the diffusion of flg22 receptor FLS2, which is relevant for the interaction of co-receptors such as BAK1 with different binding partners deciding on the signalling output balancing between growth and defence^10,62^.

For the actin filaments, although the membrane fluidity modification has induced the corresponding changes in actin organisation, their roles in the signalling pathway still need to be further characterised. Based on the localisation and appearance of actin foci, which was similar in response to the G-actin sequestering drug cytochalasin D or cold stress, it is speculated that the foci would be Actin-related protein 3 (Arp3), a core element of actin nucleation sites^63^.

In summary, from our study a model emerged that explains the rule of the membrane-microtubule fluidity circuit with harpin-elicited ETI-like immunity and flg22-triggered PTI in grapevine. The core element of this model is a bifurcation of signalling, with membrane rigidification functionally associating to calcium influx and microtubules, while membrane fluidisation functionally associating to RboH-dependent oxidative burst and actin filaments. Both signal chains are partially antagonistic, partially synergistic and contribute differentially to different sets of defence-activated transcripts (phytoalexin-synthesis versus jasmonate signalling). This model can resolve numerous seemingly paradox observations and numerous implications of this model could be confirmed by experimental data from this study. We find microtubules to work as modifiers of signalling, not as elements of signalling. Future work will continue to explore the senor role of plasma membrane-microtubule in grapevine immunity. On the one hand, it is necessary to identify the interactions between plasma membrane and microtubules via testing whether the action of substances (DMSO and BA) that modify membrane fluidity can be counteracted by microtubule drugs (oryzalin and taxol). One the other hand, possible candidates for the cross-talk between the signalling pathways are members of the MAPK signal cascades and transcription factors regulated by those cascades, whose responses are the subject of subsequent research.

## Material and methods

### Cell lines

To visualise microtubules and actin filaments in vitro, cell lines of *V. rupestris* expressing GFP in fusion with β-tubulin *AtTUB6*^64^ and *V. vinifera* cv. ‘Chardonnay’ expressing the actin-binding domain of plant fimbrin (FABD2) in fusion with GFP^65^ were used. The cells were cultivated as described previously^66^ in Murashige and Skoog medium supplemented with 3% w/v sucrose, 200 mg L^–1^ KH_2_PO_4_, 100 mg L^–1^ *myo*-inositol, 1 mg L^–1^ thiamine, and 0.2 mg L^–1^ 2,4‐dichlorophenoxyacetic acid (2,4-D) as auxin. Cells were subcultured every seven days, adding the appropriate antibiotics were added into different transgenic cell lines (30 mg L^–1^ hygromycin in the case of the tubulin marker line, 30 mg L^–1^ kanamycin in the case of the actin marker line).

### Pharmacological treatments

#### Modulation of membrane fluidity, microtubules, calcium influx, and apoplastic oxidative burst

The membrane “rigidifier” Dimethylsulphoxide (DMSO, 2% v/v; Roth, Karlsruhe, Germany), and the membrane “fluidiser” Benzylalcohol (BA, 10 mM, Roth, Karlsruhe, Germany) were used to modulate plasma-membrane fluidity^66–68^. The microtubule compounds taxol and oryzalin were employed to stabilise or disrupt microtubules, respectively^38^. Diphenyleneiodonium chloride (DPI, 100 µM; Sigma-Aldrich, Deisenhofen, Germany) was used to inhibit apoplastic oxidative burst by the plasma membrane located NADPH oxidases^69^, and Gadolinium chloride (GdCl_3_, 100 µM; Sigma-Aldrich, Deisenhofen, Germany) to block the calcium channels^66^. All inhibitors were diluted from a stock solution in DMSO, the maximal concentration of the solvent was 0.1%, and the experimental design, therefore, included one set with 0.1% DMSO as solvent control. To probe for the effect of microtubule stability, cells were treated with 10 μM taxol (Sigma-Aldrich, Deisenhofen, Germany) prior to elicitation by harpin or flg22. To eliminate microtubules, we added 10 μM of oryzalin (Sigma-Aldrich, Deisenhofen; Germany) 1 h prior to elicitation. A solvent control with 0.1% DMSO was included throughout.

### Modulation of membrane and microtubule dynamics upon elicitation

We assessed the roles of plasma membrane fluidity and microtubule dynamics during the cellular response to the bacterial elicitors. For this purpose, the *V. rupestris* GFP-TuB6 cells were pre-treated with either 2% DMSO, 10 mM BA, 10 μM taxol, and 10 μM oryzalin for half an hour. Subsequently, the pre-treated cells were elicited for 1 h with either 1 µM flg22 to induce basal immunity, or with 9 μg/ml harpin [Messenger, EDEN Bioscience Corporation, Washington, USA; active ingredient: 1% (w/w) harpin protein] to induce cell-death-related immunity. In a variation of this experiment, DMSO, BA, and oryzalin were administered not prior to, but simultaneously with, harpin to follow the development of mortality over time, scoring 12, 24, and 48 h after the treatment.

The cell line *V. vinifera* cv. ‘Chardonnay’ *FABD2-GFP* served to report the response of actin filaments. These cells were treated with either 2% DMSO, 10 mM BA or 0.1% DMSO (as solvent control) for half an hour before further incubating with 9 μg/ml harpin for 1 h. We observed the responses of actin filaments at the beginning and the end of the harpin treatment.

### Microscopy, image processing, and quantitative image analysis

The cytoskeletal responses to different chemical treatments were monitored making use of the *GFP*-*AtTuB6* marker (*V. rupestris*), or the *FABD2-GFP* marker (*V. vinifera* L. cv. ‘Chardonnay’) strains, respectively. We followed individual cells over time by spinning-disc confocal microscopy. The fluorescence of GFP was captured using a CCD camera on an AxioObserver Z1 (Zeiss, Jena, Germany) using a 63 × LCI-Neofluar Imm Corr DIC objective (NA 1.3), the 488 nm emission line of an Ar-Kr laser, and a spinning-disc device (YOKOGAWA CSU-X1 5000). To operate imaging, we used the ZEN 2012 (Blue edition) software platform to generate orthogonal projections from the recorded stacks, and to export the raw images in TIFF format. For each experimental set, representative images of at least three independent experimental series recording a population of 30 individual cells.

To quantify microtubule integrity, we adopted a strategy from Schwarzerová et al.^70^ using the freeware ImageJ (https://imagej.net)^70^. Four intensity profiles were collected along the elongation axis (i.e. perpendicular to MTs) in equal spacing along the cross axis (using the shortcut Ctrl+K). The probing line was 25-pixel width to minimise the impact of random noise. After the import of the profiles into a preformed Excel sheet, the first derivatives were calculated. Subsequently, we added each value with the value from the preceding position of the profile to filter out fluctuations from background noise that were not deriving from a microtubule. The standard deviation over these filtered first derivatives increases proportionally with the steepness of the landscape, i.e. with the integrity of microtubules. To normalise for different background levels (that might arise either from diffuse tubulin fluorescence from non-assembled tubulin, or from differences in image acquisition parameters), this standard deviation was put into relation to the maximal intensity within the profile. Values represent between 12 and 20 individual cells per treatment.

### Extracellular alkalinisation

Extracellular alkalinisation was measured by combining a pH meter (Schott handy lab, pH 12) with a pH electrode (Mettler Toledo, LoT 403-M8-S7/120), and recorded by a paperless readout (VR06; MF Instruments GmbH, Albstadt-Truchtelfingen, Germany). Prior to the addition of chemicals, we equilibrated the cells on an orbital shaker for at least 1 h. Data represent at least five independent experimental series.

### Quantification of cytosolic calcium levels

Chloro-tetracycline was used to report the cytosolic calcium elicited by various treatments^44^. The cells were either treated with water control, 10 mM BA, 2% DMSO, 10 mM BA, 9 µg/ml harpin, a combination of harpin and BA, or the combination of harpin and DMSO for 25 min. After collecting, the cells were fixed in 2.5% glutaraldehyde in 200 mM sodium phosphate buffer (pH 7.4). After transferring into the custom-made staining chamber, the cells were washed with 50 mM of staining buffer (Tris-HCl, pH 7.45) for three times, each wash procedure continues 5 min. After draining, the filter paper was used to remove the excess liquid. The cells were stained with 100 μM chlorotetracycline for 5 min in 5-ml beakers in staining buffer. Unbound dye was washed out three times with staining buffer for 2 min and then analysed by spinning-disc confocal microscopy. To analyse the green fluorescence distribution in a quantitative manner, the skewness of the intensity histogram of the images was used as a readout according to Wang et al.^45^.

### RNA extraction and quantitative RT-PCR analysis

Total RNA was isolated using the Universal RNA Purification Kit (Roboklon, Germany). We used the optional on-column DNase (Qiagen, Hilden, Germany) digestion was conducted as defined in the protocol of the producer. Quantity and quality of the obtained RNA were checked spectrophotometrically (NanoDrop, Radnor, USA), and, in parallel, by electrophoresis on a 0.8% agarose gel. For reverse transcription of the mRNA into cDNA using the M-MuLV cDNA Synthesis Kit (New England Biolabs; Frankfurt am Main, Germany) followed the instructions of the manufacturer. The amount of RNA template was adjusted to 1 μg.

After quantitative RT-PCR (qPCR) using a CFX96^TM^ real-time PCR cycler (Bio-RAD, USA) expression levels of target genes were calculated with the 2^-ΔΔCt^ method^71^ and normalised to elongation factor 1 (*EF1-α*) as a house-keeping gene. Genes involved in grapevine basal immunity such as the phenylpropane phytoalexin synthesis genes phenylalanine ammonia lyase (PAL), stilbene synthase (StSy), and resveratrol synthase (RS), the transcription factor MYB14 as regulator of stilbene synthesis, and the jasmonate ZIM/tify-domain protein 1 (JAZ1) as a readout for jasmonate signalling were used. Accession numbers of these genes and the primer details are given in Table S1. Each experiment represents three biological replicates, each done as technical triplicate.

### Determination of cell mortality

To determine mortality, the Evans Blue dye exclusion test was used^72^. For each sample, aliquots of 200 μL cells were transferred into custom-made staining chambers, to remove the medium. After staining for 3 min in 2.5% (w/v) Evans Blue, the cells were washed with distilled water several times. Aliquots of 40 μL stained cells were observed under an AxioImager Z.1 microscope (Zeiss, Jena) equipped with an ApoTome microscope slider through the filter sets 38 HE. Evans Blue penetrates dead cells resulting in blue staining of the cell. Each measurement consisted of at least 1000 scored cells. Data represent a population of 3000 cells scored over three independent experiments.

## Supplementary information

Supplemental data

## Data Availability

Data are available upon request to the corresponding author.
