# Supplementary material for: Dissecting the membrane-microtubule sensor in grapevine defence
Source: Hortic Res. 2021 Dec 1;8:260. doi: 10.1038/s41438-021-00703-y (PMC8632924; doi:10.1038/s41438-021-00703-y)
Supplement: Supplementary file 1 — Supplemental data [file 41438_2021_703_MOESM1_ESM.doc]

**Dissecting the Membrane-Microtubule Sensor in Grapevine Defence**

Pingyin Guan^1,2*^, Wenjing Shi^2^, Michael Riemann^2^, Peter Nick^2*^

1. College of Horticulture, China Agricultural University, Beijing 100193, China

2. Molecular Cell Biology, Botanical Institute, Karlsruhe Institute of Technology, Fritz-Haber-Weg 4, 76131 Karlsruhe, Germany

*authors for correspondence:

Pingyin Guan, College of Horticulture, China Agricultural University, Beijing 100193, China

*Tel: +86 10-62732488, Email:* [*pyguan@cau.edu.cn*](mailto:pyguan@cau.edu.cn)

Peter Nick, Molecular Cell Biology, Botanical Institute, Karlsruhe Institute of Technology, Fritz-Haber-Weg 4, 76131 Karlsruhe, Germany.

*Tel: +49 721 608 42142, Fax: +49 721 608 44193, Email: peter.nick@kit.edu*

Pingyin Guan: [pyguan@cau.edu.cn](mailto:pyguan@cau.edu.cn).

Michael Riemann: [michael.riemann@kit.edu](mailto:michael.riemann@kit.edu).

Wenjing Shi: ex3817@partner.kit.edu.

Peter Nick: [peter.nick@kit.edu](mailto:peter.nick@kit.edu).

Short title: Membrane fluidity regulates grapevine defence

**Supplementary data**

**
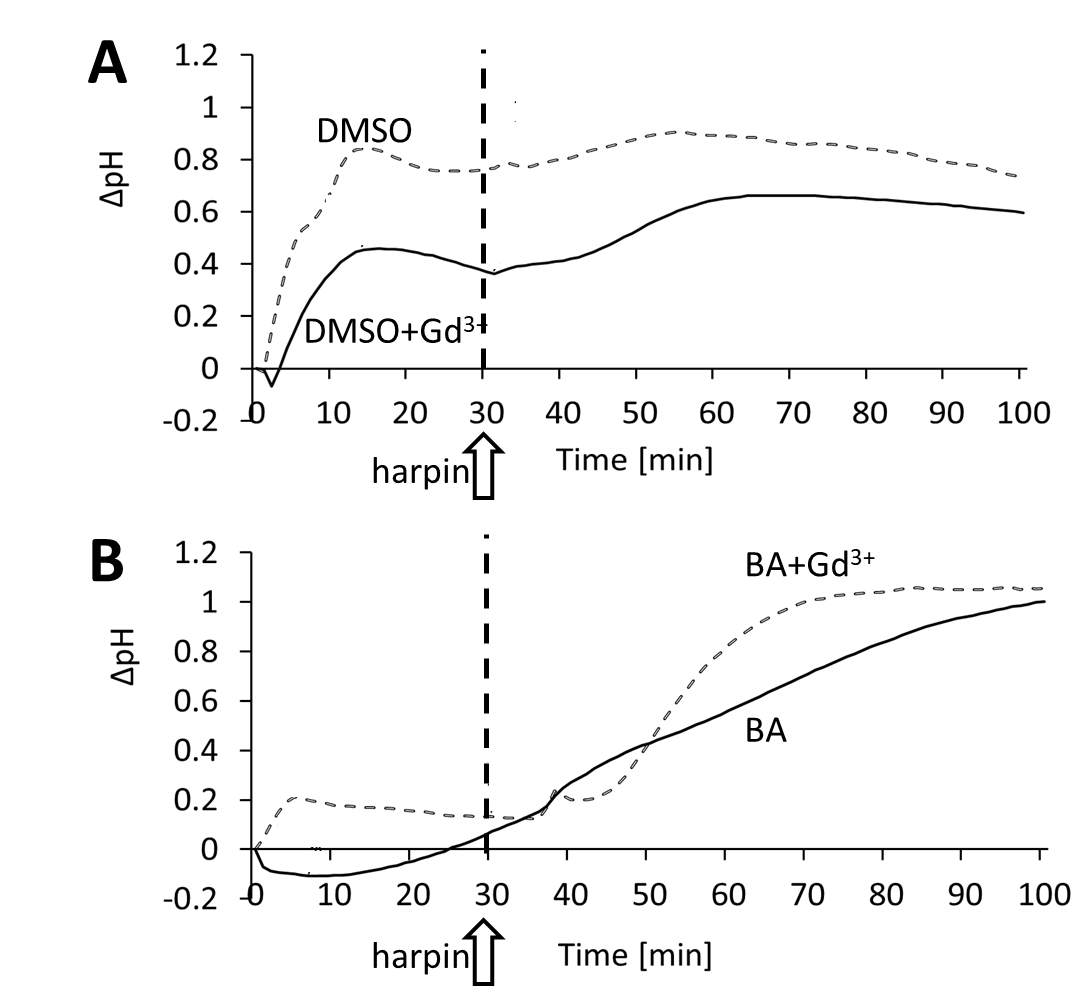
**

**Fig. S1** Extracellular alkalinisation induced by harpin (9 µg/ml) in combination withDMSO (2%, **A**) and BA (10 mM, **B**) after pre-treatment with 100 μM GdCl_3_ for half an hour.


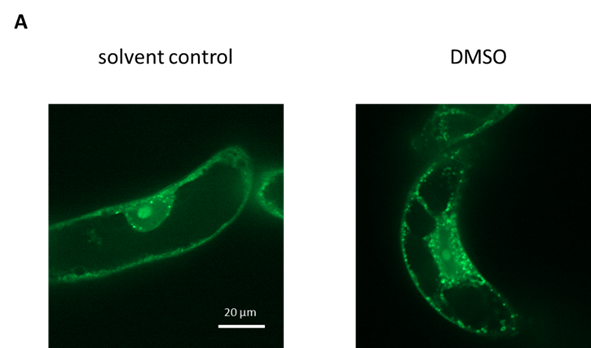


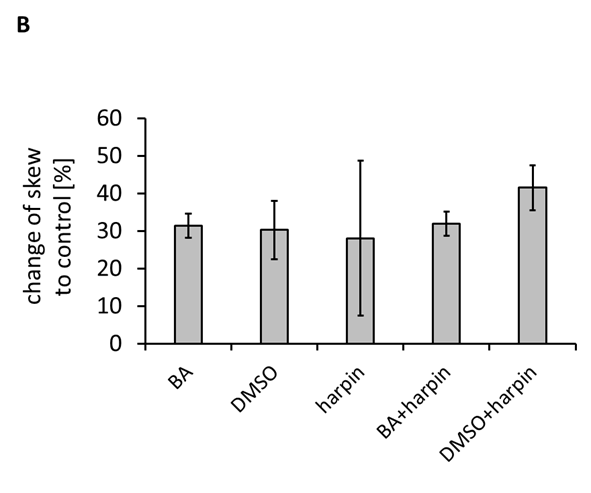


**Fig. S2** Effect of various chemicals on intracellular calcium levels reported by chloro-tetracycline. A Representative example cell either treated with solvent water or 2% DMSO for 25 min. Size bar corresponds to 20 μm. B Change of a skew on cells either 10 mM BA, 2% DMSO, 10 mM BA, 9 µg/ml harpin, a combination of harpin and BA, or the combination of harpin and DMSO for 25 min. The change of skewness was determined via comparing the skewness of various chemical treated cells and water-treated cells. Data represent mean values and standard errors from at least three independent biological replicates. Statistical significance was assessed using a student t-test.


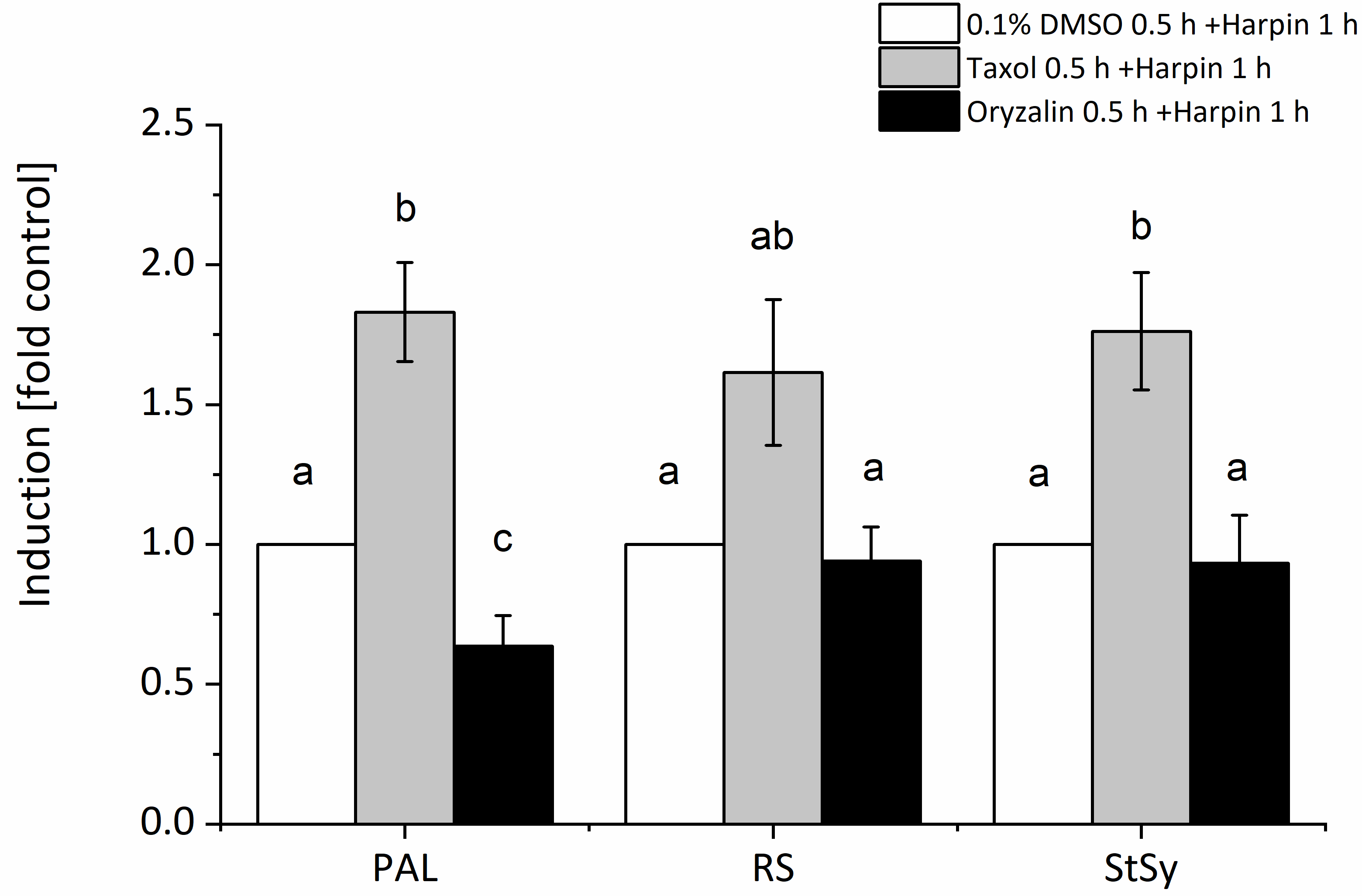


**Fig. S3** Effect of taxol and oryzalin on steady-state transcript levels of phytoalexins-synthesis genes *PAL*, *RS*, and *StSy* in response to harpin (9 µg/ml, 1 h) in cells of *V. rupestris*. The cells were pre-treated with either 0.1% DMSO (solvent control), 10 μM taxol or 10 μM oryzalin for half an hour. Transcripts were measured against Elongation Factor 1*-α* (EF1*-α*) as internal standard for quantification. Data represent means and standard error from three biological replicates with three technical replicates each. Means denoted by different letters are significantly different at *P* < 0.05.

**Table S1.** List of oligonucleotide primers used for expression analysis by quantitative PCR.

*EF1-α*, elongation factor 1-*α*; *PAL*, phenylalanine ammonia lyase 1; *RS*, resveratrol synthase; *StSy*, stilbene synthase; *MYB14*, stilbene biosynthesis related the specific transcription factor; *JAZ1*, jasmonate ZIM/tify-domain protein 1 (JAZ1).

| **Name** | **GenBank accession no.** | **Primer sequence 5'-3'** | **Reference** |
| --- | --- | --- | --- |
| EF1α | EC959059 | Sense: 5'-GAACTGGGTGCTTGATAGGC-3'  Antisense: 5’-AACCAAAATATCCGGAGTAAAAGA-3’ | [1] |
| PAL | X75967 | Sense: 5’-TGCTGACTGGTGAAAAGGTG-3’  Antisense: 5’-CGTTCCAAGCACTGAGACAA-3’ | [2] |
| RS | AF274281 | Sense: 5'-TGGAAGCAACTAGGCATGTG-3'  Antisense: 5'-GTGGCTTTTTCCCCCTTTAG-3' | [3] |
| StSy | X76892 | Sense: 5‘-CCCAATGTGCCCACTTTAAT-3’  Antisense: 5'-CTGGGTGAGCAATCCAAAAT-3' | [3] |
| MYB14 | NW_003724037.1 | Sense: 5′-CTACTGACGTGCACTAGCCT-3′  Antisense: 5′-GCAG AGTGAAAGTGCAACACG-3’ | [3] |
| JAZ1 | JF900329 | Sense: 5’-TGCAGTCTGTTGAGCCAATACATA-3’  Antisense: 5’-CACGTTTCCGGACTTCTTTACAC-3’ | [4] |

References

1 Reid, K. E., Olsson, N., Schlosser, J., Peng, F. & Lund, S. T. An optimized grapevine RNA isolation procedure and statistical determination of reference genes for real-time RT-PCR during berry development. *BMC plant biology* **6**, 1-11 (2006).

2 Belhadj, A. *et al.* Effect of methyl jasmonate in combination with carbohydrates on gene expression of PR proteins, stilbene and anthocyanin accumulation in grapevine cell cultures. *Plant Physiology and Biochemistry* **46**, 493-499 (2008).

3 Duan, D. *et al.* Genetic diversity of stilbene metabolism in *Vitis sylvestris*. *Journal of Experimental Botany* **66**, 3243-3257 (2015).

4 Ismail, A., Riemann, M. & Nick, P. The jasmonate pathway mediates salt tolerance in grapevines. *Journal of Experimental Botany* **63**, 2127-2139 (2012).
